# Development of a Risk Score for the Prediction and Management of Pre-Eclampsia in Low-Resource Settings

**DOI:** 10.3390/jcm14103398

**Published:** 2025-05-13

**Authors:** Victor Bogdan Buciu, Dorin Novacescu, Flavia Zara, Denis Mihai Șerban, Larisa Tomescu, Sebastian Ciurescu, Sebastian Olariu, Marina Rakitovan, Antonia Armega-Anghelescu, Alexandu Cristian Cindrea, Mihai Ionac, Veronica-Daniela Chiriac

**Affiliations:** 1 Doctoral School, “Victor Babes” University of Medicine and Pharmacy Timisoara, E. Murgu Square, No. 2, 300041 Timisoara, Romania; victor.buciu@umft.ro (V.B.B.); sebastian.ciurescu@umft.ro (S.C.); raul.olariu@umft.ro (S.O.); marina.rakitovan@umft.ro (M.R.); antonia.armega@umft.ro (A.A.-A.); alexandru.cindrea@umft.ro (A.C.C.); 2 Department II of Microscopic Morphology, “Victor Babes” University of Medicine and Pharmacy Timisoara, E. Murgu Square, No. 2, 300041 Timisoara, Romania; flavia.zara@umft.ro; 3 Department of Obstetrics-Gynaecology, Discipline of Obstetrics-Gynecology, “Victor Babes” University of Medicine and Pharmacy Timisoara, E. Murgu Square, No. 2, 300041 Timisoara, Romania; denis.serban@umft.ro (D.M.Ș.); tomescu.larisa@umft.ro (L.T.); chiriac.veronica@umft.ro (V.-D.C.); 4 Department of Microsurgery, Vascular Surgery and Scientific Research Methodology, “Victor Babes” University of Medicine and Pharmacy Timisoara, E. Murgu Square, No. 2, 300041 Timisoara, Romania; mihai.ionac@gmail.com

**Keywords:** hypertensive disorders of pregnancy, pre-eclampsia, maternity, postpartum, predictive score, cardiovascular disease, medical, diagnostic and prognostic error, maternal–fetal medicine, high blood pressure, clinical management

## Abstract

**Background:** Pre-eclampsia is a significant hypertensive disorder affecting 2–8% of pregnancies globally, significantly contributing to maternal/perinatal deaths. Early identification of at-risk patients is crucial for reducing these mortalities, yet first-trimester screening remains inaccessible in many low-resource settings. This study aims to develop a second-trimester risk stratification model based on clinical parameters to assist in managing pre-eclampsia in diverse healthcare contexts. **Methods:** This retrospective cohort study analyzed medical records from 700 pregnancies (350 with preeclampsia, 350 controls) between January 2021 and August 2024 at a tertiary medical center in western Romania. Sample size was calculated to achieve 90% power with α = 0.05 for detecting clinically significant differences between groups. Data analysis focused on clinical variables such as maternal age, hypertension, diabetes, and socioeconomic factors. A scoring model was developed using logistic regression and validated for predictive accuracy using ROC curve analysis, with AUC as the primary metric. Calibration was assessed using the Hosmer–Lemeshow test. **Results:** The risk stratification model demonstrated an AUC of 0.91 (95% CI: 0.88–0.94), indicating high discriminative capability. The model showed good calibration (*p* = 0.78). Sensitivity was 74.4%, and specificity reached 97.8%. Patients were categorized into low (0–4 points), moderate (5–7 points), and high-risk (≥8 points) groups based on optimized cut-off values. High-risk patients showed significantly higher rates of adverse outcomes, including eclampsia (12.3% vs. 0% in low-risk, *p* < 0.001) and HELLP syndrome (8.7% vs. 0.5% in low-risk, *p* < 0.001). Neonates born to high-risk mothers had lower birth weight (mean difference: 486 g, *p* < 0.001), smaller head circumference (mean difference: 2.3 cm, *p* < 0.001), and lower APGAR scores (median difference: 2 points, *p* < 0.001). **Conclusions:** This novel model offers a practical second-trimester risk assessment tool that leverages routine clinical data available after 20 weeks of gestation. It facilitates targeted care and resource allocation, particularly benefiting settings lacking early screening access. Implementation of risk-stratified management protocols could significantly improve maternal and neonatal outcomes in diverse healthcare environments.

## 1. Introduction

Pre-eclampsia is a complex hypertensive disorder affecting 2–8% of pregnancies worldwide, contributing substantially to maternal and perinatal morbidity and mortality rates globally [1]. With an estimated 76,000 maternal deaths and 500,000 perinatal deaths annually attributable to pre-eclampsia and related hypertensive disorders, the condition represents one of the leading preventable causes of maternal mortality, particularly in low and middle-income countries [2]. Defined by the onset of hypertension, often accompanied by proteinuria, after the 20th week of gestation, pre-eclampsia results from an intricate interaction between genetic, immunologic, and environmental factors. These factors ultimately disrupt placental perfusion and induce systemic endothelial dysfunction, affecting multiple organ systems and increasing the risk of adverse outcomes [3,4].

The pathophysiology of pre-eclampsia, though incompletely understood, centers around abnormal placental development and function, resulting in widespread maternal endothelial dysfunction [5]. This dysfunction is exacerbated by poor placentation, leading to a dysregulated release of anti-angiogenic factors like soluble fms-like tyrosine kinase-1 (sFlt-1) and pro-angiogenic factors such as placental growth factor (PlGF). Together, these imbalances contribute to hypertension, systemic inflammation, and organ damage in the mother [6,7].

Recognizing high-risk patients in the first trimester is essential to enable timely intervention. However, in many healthcare systems—especially in low- and middle-income regions—a substantial proportion of pregnant women first present after 20 weeks of gestation, missing the window for first-trimester screening and preventive aspirin therapy. This delayed presentation creates an urgent need for alternative risk stratification tools applicable later in gestation. Current predictive models incorporate maternal history, biochemical markers (e.g., PlGF, pregnancy-associated plasma protein-A (PAPP-A)), and biophysical parameters like uterine artery Doppler indices, which have shown promise in identifying early onset pre-eclampsia [8]. When these early screening tools are accessible, preventive measures, including low-dose aspirin initiated by 12–16 weeks, can reduce pre-eclampsia incidence by 62% in high-risk groups and 30% across all pregnancies, offering a significant opportunity for improved outcomes [9].

However, in low-resource settings, where access to first-trimester screening may be limited by geographic, economic, or healthcare system constraints, or in patients who present later in pregnancy, alternate approaches for risk stratification are needed. These circumstances are particularly prevalent among underserved populations, which face barriers to early prenatal care due to socioeconomic and healthcare access disparities [10]. Statistics show that, in many developing regions, up to 40% of pregnant women receive no prenatal care before the second trimester, making early interventions impossible without alternative assessment methods [11].

The primary objective of this study was to develop a second-trimester clinical risk score, based solely on routinely available variables, to predict the likelihood of pre-eclampsia. A secondary objective was to examine the associated maternal and neonatal outcomes across different risk levels. This model is particularly intended for optimal use between 20 and 24 weeks of gestation, in settings wherein early biomarker-based screening is not feasible. We also discuss how stratification at this stage may support clinical decision-making through targeted monitoring, antihypertensive therapy, fetal surveillance, and delivery planning in high-risk patients. Unlike existing models that rely on specialized biochemical testing or advanced imaging, our approach utilizes parameters readily available in standard clinical settings. Although not optimal, due to even more limited prospective interventions, the model is applicable after 24 weeks of gestation, making it especially useful for patients who first access prenatal care late in pregnancy, which is common in low-resource settings.

## 2. Materials and Methods

### 2.1. Study Population

This study retrospectively reviewed medical records from 700 patients treated at our tertiary care center in western Romania between January 2021 and August 2024. Our institution manages approximately 4500–5000 deliveries annually. Over the study period, more than 1100 cases of pre6eclampsia were identified, from which 350 were randomly selected to ensure statistical power. A 1:1 ratio of controls (*n* = 350) was selected based on matched age and delivery timing to minimize confounding. The sample size was determined using power analysis, calculating that 350 cases per group would provide 90% power at α = 0.05 to detect a clinically significant difference in outcomes, assuming a moderate effect size (Cohen’s d = 0.4). Among the participants, 350 had been diagnosed with pre-eclampsia according to the American College of Obstetricians and Gynecologists (ACOG) criteria, while 350—the control group—had uncomplicated pregnancies matched for age and delivery timing.

Participants were eligible for inclusion if they were over 18 years old, had completed delivery at our institution, and either had a confirmed diagnosis of pre-eclampsia (according to ACOG criteria) or had an uncomplicated pregnancy. We excluded patients with chronic renal disease (serum creatinine > 1.2 mg/dL), significant cardiovascular disorders (e.g., cardiomyopathy, symptomatic valvular disease, or coronary artery disease), autoimmune diseases requiring immunosuppression, hepatic dysfunction, or known thrombophilia. These exclusions ensured that other underlying conditions did not confound pre-eclampsia outcomes. The study was conducted in accordance with the Declaration of Helsinki and approved by the Institutional Review Board of the Municipal Emergency Clinical Hospital and Victor Babes University of Medicine and Pharmacy Timisoara (protocol code 78/02.10.2023). Informed consent was obtained from all subjects involved in the study. Patient data were anonymized and handled according to institutional privacy protocols. To reduce potential bias, data abstraction was performed independently by two trained researchers. Discrepancies were resolved by a third reviewer. Controls were selected using predefined matching criteria, and multivariable logistic regression was used to adjust for known confounders.

### 2.2. Data Collection and Predictive Model Development

Data were compiled and analyzed using Microsoft Excel 2016 and SPSS version 26. We identified key clinical variables known to correlate with pre-eclampsia, including maternal hypertension, diabetes, maternal age, parity, educational level, and insurance status. For defining hypertension, we applied the ACOG criteria, where hypertension was defined as blood pressure readings of ≥140/90 mmHg on two occasions at least 4 h apart, or the presence of antihypertensive treatment [12]. Diabetes was diagnosed based on fasting plasma glucose levels ≥ 126 mg/dL, or positive oral glucose tolerance test results, consistent with American Diabetes Association guidelines [13].

The scoring system assigned points to each clinical predictor based on its correlation with pre-eclampsia risk, determined through logistic regression analysis. Variables were weighted according to their adjusted odds ratios, with statistically significant predictors receiving higher weights. Gestational age beyond 37 weeks, current hypertension, and diabetes were weighted more heavily in the scoring algorithm (3 points each) given their high statistical relevance in prior research [14]. Maternal age > 35 years, nulliparity, lower educational attainment, and absence of health insurance were assigned lower weights (1–2 points) based on their respective adjusted odds ratios.

The cut-off points for risk categories (0–4 points for low risk; 5–7 points for moderate risk; ≥8 points for high risk) were determined using the Youden Index to optimize the balance between sensitivity and specificity. These thresholds were validated through bootstrap resampling with 1000 iterations to ensure stability.

Cases with more than 10% missing data on key variables were excluded from the analysis. For variables with less than 5% missingness, single imputation using the median (for continuous variables) or mode (for categorical variables) was applied.

### 2.3. Model Validation and Outcome Analysis

To assess the model’s accuracy, it was applied retrospectively to our cohort, comparing risk scores with observed maternal and neonatal outcomes. Primary fetal outcome measures included birth weight, head circumference, thorax circumference, fetal length, and APGAR scores. Maternal outcomes were also documented, including the rate of severe complications such as eclampsia or hemolysis, elevated liver enzymes, and low platelet count (HELLP) syndrome, postpartum hemorrhage, and need for intensive care admission.

Statistical analysis was conducted using SPSS Version 26 and utilized descriptive methods and non-parametric tests (Kruskal–Wallis) to compare outcomes across risk levels. Differences between categorical variables were assessed using Chi-square or Fisher’s exact tests as appropriate. ROC curve analysis evaluated the model’s discriminative accuracy, with the area under the curve (AUC) serving as the primary metric. Model calibration was assessed using the Hosmer–Lemeshow goodness-of-fit test, with *p* > 0.05 indicating adequate calibration.

## 3. Results

### 3.1. Baseline Characteristics

The baseline demographic and clinical characteristics of the study population are presented in Table 1. Women who developed pre-eclampsia were more likely to be older, nulliparous, and have comorbidities such as chronic hypertension and diabetes. Significant differences were also observed in socioeconomic factors, with lower educational attainment and lack of health insurance being more prevalent in the pre-eclampsia group.

### 3.2. Risk Score Development

**Figure 1:**
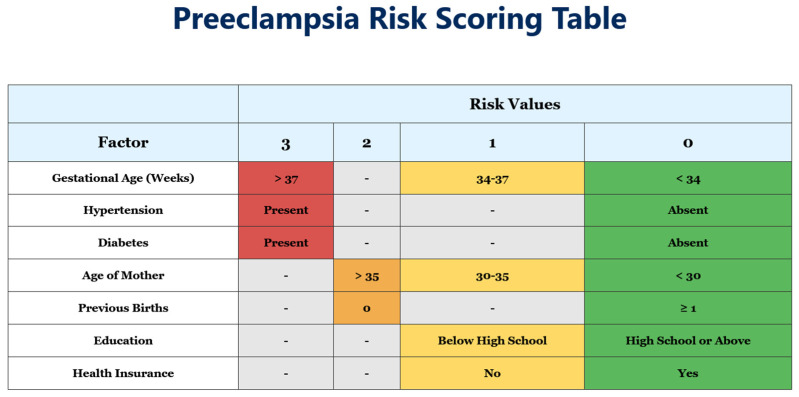
Clinical risk scoring system for pre-eclampsia prediction in the second trimester. The figure above emphasizes the weight each individual status would add up to the final risk score, with color-coding three points, two points, one point and 0 points respectively to red, orange, yellow and green.

When applied to the cohort of 700 pregnancies, the second-trimester risk scoring model demonstrated strong overall performance. As seen in Figure 1, the risk scoring system assigns points based on clinical factors assessed in the second trimester. Each factor is weighted according to its association with pre-eclampsia development. Higher point totals indicate increased risk. Gestational age > 37 weeks, presence of hypertension, and presence of diabetes each receive the highest weighting (3 points) based on their strong predictive value. The risk score was designed to be applied starting at 20 weeks of gestation and is intended for use at any point thereafter during the pregnancy, based on available clinical data at the time of presentation. Although not optimal, as discussed further on, we have highlighted the applicability of such tools in our socioeconomic status.

**Figure 2:**
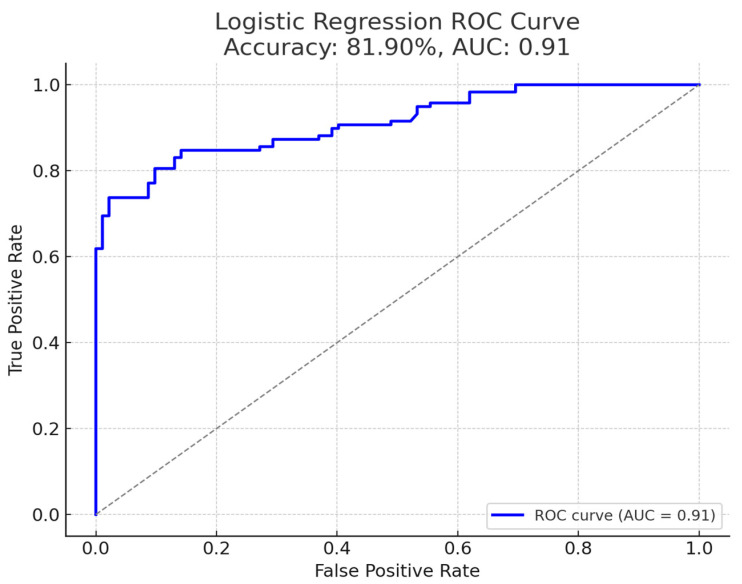
Logistic regression ROC curve demonstrating the predictive performance of the pre-eclampsia risk model. With an area under the curve (AUC) of 0.91 and overall accuracy of 81.90%, the model shows high discriminative capability. The curve illustrates the relationship between true positive rate (sensitivity) and false positive rate (1-specificity), with the optimal threshold providing 74.4% sensitivity and 97.8% specificity.

As seen in Figure 2, the model achieved an accuracy of 81.9% for predicting pre-eclampsia, with an area under the ROC curve (AUC) of 0.91 (95% CI: 0.88–0.94), indicating high discriminative capability. The Hosmer–Lemeshow test yielded *p* = 0.78, indicating good calibration. At the chosen risk score threshold, the sensitivity was 74.4% and specificity was 97.8%. This high specificity reflects a low false-positive rate in identifying at-risk cases, while the moderate sensitivity suggests some pre-eclamptic cases were not captured by the model’s cut-off.

**Figure 3:**
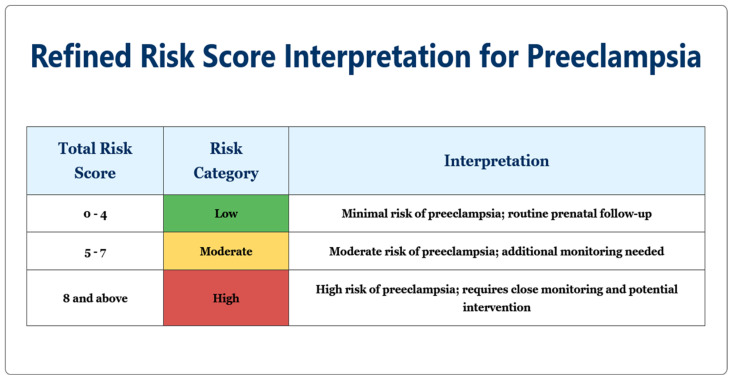
Clinical interpretation of the total risk scores for pre-eclampsia. Patients are categorized into three risk groups: low risk (0–4 points) requiring routine prenatal follow-up, moderate risk (5–7 points) requiring additional monitoring, and high risk (≥8 points) requiring close monitoring and potential intervention. The color-coded format (green, yellow, red) provides visual stratification of risk levels.

Using the scoring system, each patient was categorized into one of three risk groups based on their total score, as seen in Figure 3: low risk (0–4 points), moderate risk (5–7 points), or high risk (≥8 points). Risk categorization was applied to all 700 study participants (both pre-eclampsia and control groups) using this newly developed scoring system, in order to demonstrate its predictive value for maternal and neonatal outcomes, as seen below.

### 3.3. Maternal Outcomes by Risk Category

A clear gradient in maternal outcomes was observed across the stratified risk groups (see Table 2). Women in the low-risk group rarely experienced severe pre-eclampsia and had generally favorable outcomes under standard prenatal care. Their blood pressure typically remained stable throughout pregnancy, and the incidence of complications was minimal.

In contrast, the high-risk group showed a markedly higher incidence of adverse maternal outcomes. These high-risk patients had significantly increased rates of severe pre-eclampsia and related complications, including occurrences of eclampsia, whereas no cases of eclampsia were noted among low-risk patients (*p* < 0.001 for group difference). High-risk mothers were also more likely to develop severe hypertension requiring intervention and had higher rates of ICU admission and emergency cesarean delivery.

The moderate-risk group exhibited an intermediate profile: while most moderate-risk pregnancies did not progress to severe disease, this group required closer surveillance. Some moderate-risk women benefited from prophylactic measures (such as low-dose aspirin), which appeared to mitigate the progression of pre-eclampsia symptoms in those cases. Maternal blood pressures in the moderate group were mildly elevated but generally well-managed, and the frequency of severe complications was between that of the low- and high-risk groups.

### 3.4. Neonatal Outcomes by Risk Category

**Figure 4:**
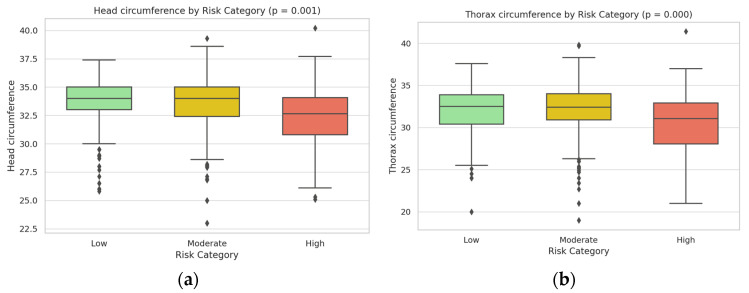
Dual-panel box plot of fetal anthropometric parameters by risk category: (**a**) Head circumference measurements across the three risk categories, demonstrating a significant decreasing trend (*p* = 0.001) from low to high-risk groups; (**b**) Thorax circumference measurements across risk categories, also showing significant reduction (*p* = 0.001) in the high-risk group compared to low and moderate-risk groups.

Neonatal outcomes mirrored the risk stratification, showing significant trends in fetal growth and well-being across the three groups (Table 3). Infants born to low-risk mothers had the highest birth weights and overall healthy neonatal indices, whereas those in the high-risk category had markedly reduced birth weights on average, consistent with an increased incidence of fetal growth restriction in pregnancies complicated by severe pre-eclampsia (see Figure 4a).

**Figure 5:**
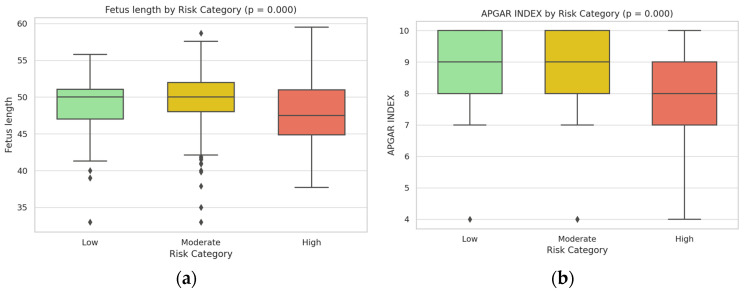
Dual-panel box plot of fetal length and APGAR scores by risk category: (**a**) Fetal length measurements across the three risk categories, showing a significant decreasing trend (*p* = 0.000) from low to high-risk groups; (**b**) APGAR index scores across risk categories, demonstrating significantly lower values (*p* = 0.000) in the high-risk group.

High-risk group neonates had significantly lower mean birth weight and smaller head and thorax circumference at birth compared to neonates in the low-risk group (*p* < 0.001) (see Figure 4a,b). The proportion of low-birth-weight infants (<2500 g) was highest in the high-risk category, indicating an adverse impact of maternal risk factors on fetal growth (see Figure 5a). APGAR scores followed a similar downward trend with increasing maternal risk (see Figure 5b). Five-minute APGAR scores were within normal ranges for nearly all low-risk infants (median scores around 9), whereas high-risk infants had appreciably lower APGAR scores on average (with median values closer to 7–8, indicating more neonates requiring resuscitative attention). The moderate-risk group’s neonatal outcomes were intermediate: these infants showed slight decreases in birth weight and APGAR scores relative to the low-risk group, but most remained within clinically normal limits.

All differences in key outcomes between the risk categories were statistically significant. In particular, comparison of the groups demonstrated highly significant overall effects of risk stratification on maternal complication rates and neonatal health indicators (*p* < 0.001 for trend across low, moderate, and high-risk groups). Box plot analyses of the neonatal anthropometric measures and APGAR scores by risk group (Figure 4 and Figure 5) visually underscored these findings, illustrating a stepwise decline in birth weight and neonatal size metrics from the low-risk through high-risk categories. The high-risk group’s distributions showed minimal overlap with those of the low-risk group, confirming that higher maternal risk scores were strongly associated with worse neonatal outcomes. These results clearly indicate that the risk score effectively stratified patients such that higher second-trimester risk scores correspond to a significantly elevated likelihood of severe maternal complications and compromised neonatal health.

### 3.5. Subgroup Analysis

Further analysis revealed some variations in the model’s performance across different demographic subgroups (Table 4). The model performed better in nulliparous women (AUC 0.93, 95% CI: 0.90–0.96) compared to multiparous women (AUC 0.88, 95% CI: 0.84–0.92), possibly reflecting the more complex interplay of risk factors in women with previous pregnancies. Similarly, performance varied slightly by age group, with better discrimination in women under 35 years (AUC 0.92, 95% CI: 0.89–0.95) compared to those over 35 (AUC 0.89, 95% CI: 0.85–0.93).

Subgroup performance was assessed by parity, maternal age, educational level, and insurance status. AUC values were compared using the DeLong test. Interaction terms were not included in the logistic model due to limited power and to preserve model interpretability.

These findings suggest that, while the model works effectively across all demographic groups, certain adjustments might be considered for specific populations to optimize prediction accuracy further.

## 4. Discussion

The findings of this study underscore the value of a clinically accessible, second-trimester pre-eclampsia risk stratification model, particularly in healthcare settings where early biomarker-based screenings are not feasible. By leveraging commonly available clinical factors, this model offers a pragmatic approach to categorizing patients by risk level, thereby enabling tailored follow-up care that aligns with best practices in pre-eclampsia management.

### 4.1. Clinical Relevance and Utility of the Predictive Model

The model’s high specificity (97.8%) is noteworthy, as it minimizes the likelihood of false positives, which is critical in managing resource allocation in clinical settings. This level of accuracy suggests that the model can effectively reduce unnecessary monitoring and interventions in low-risk cases, thereby allowing healthcare providers to concentrate resources and care on moderate/high-risk patients who are more likely to benefit from targeted preventive measures, such as low-dose aspirin or intensive fetal monitoring. In high-resource settings, early screening often includes angiogenic and anti-angiogenic biomarkers, such as PlGF and the sFlt-1/PlGF ratio, which have been shown to improve predictive accuracy in the first trimester [6,7]. However, these tests require specialized equipment and trained personnel, posing barriers in low-resource settings. As shown in Table 5, our model compares favorably with existing prediction tools, particularly when considering its accessibility. The simplicity and accessibility of the predictive model in this study make it a valuable tool for clinical environments where access to early biomarker testing is limited.

The model presented in this study complements existing first-trimester screening approaches by providing a reliable second-trimester alternative, particularly for patients who lack early prenatal care. While first-trimester markers like PlGF, PAPP-A, and Doppler indices provide sensitive early risk assessments, they are often inaccessible in low-resource settings [16,17,18]. Studies have demonstrated that these biomarkers, while effective, do not always yield practical application outside of well-resourced clinical environments [20]. The model in this study, by contrast, relies on clinical factors typically recorded in standard prenatal visits, making it especially relevant for late-presenting pregnancies. Its reliance on data available at or after 20 weeks of gestation fills a critical gap in pre-eclampsia risk assessment and offers a viable strategy for healthcare providers operating in diverse settings. Although designed for second-trimester use, the model’s reliance on simple clinical variables makes it potentially adaptable for earlier use in the first trimester or even during preconception counseling. Future studies may explore its predictive value across various gestational ages and clinical settings.

Pre-eclampsia is increasingly recognized as a heterogeneous condition with multiple potential etiologies, including placental, inflammatory, and cardiovascular pathways. This diversity may influence the performance of predictive models. While our score demonstrates high discriminative accuracy overall, it may be more sensitive to certain subtypes of pre-eclampsia, such as those with metabolic risk factors. This limitation underscores the need for future refinements that consider PE subtypes and their unique pathophysiologic profiles.

### 4.2. Implications for Maternal and Neonatal Outcomes

The association between pre-eclampsia and adverse neonatal outcomes, including reduced fetal growth and lower APGAR scores, observed in this study aligns with extensive research on the impact of hypertensive disorders on fetal development. Studies have consistently demonstrated that pre-eclampsia, particularly when severe or early onset, impairs placental blood flow, which can result in fetal growth restriction and preterm birth [21,22]. Reduced fetal growth parameters observed in the high-risk group may reflect placental insufficiency, a common pathophysiological feature of pre-eclampsia, where reduced placental blood flow limits nutrient and oxygen delivery to the fetus. This correlation was evident in head and thorax circumference differences, which suggest that higher maternal pre-eclampsia risk scores are predictive not only of maternal complications but also of compromised neonatal health outcomes.

The striking differences in neonatal outcomes between risk categories highlight the potential clinical value of this risk stratification tool. By identifying pregnancies at highest risk, interventions can be targeted to potentially mitigate the severity of both maternal and neonatal complications, even when early preventive measures like aspirin therapy are no longer feasible.

### 4.3. Socioeconomic Determinants and Healthcare Disparities

The inclusion of sociodemographic variables, such as education level and insurance status, reflects the influence of socioeconomic determinants on pre-eclampsia risk. Our data showed that women with lower educational attainment had a 2.5-fold increased risk of developing pre-eclampsia (OR 2.57, 95% CI: 1.82–3.63), while those without insurance had a 2.7-fold increased risk (OR 2.72, 95% CI: 1.79–4.15).

Multiple studies have shown that limited access to healthcare and lower socioeconomic status are associated with higher incidences of hypertensive disorders in pregnancy [10]. These factors can influence a woman’s likelihood of receiving early prenatal care, which in turn impacts her pre-eclampsia risk profile. Moreover, emerging evidence suggests that women from lower socioeconomic backgrounds have up to three times higher risk of pre-eclampsia complications compared to those from higher socioeconomic groups [23].

Additionally, healthcare accessibility and insurance coverage play crucial roles in a patient’s capacity to undergo regular prenatal screenings and access preventive interventions, such as low-dose aspirin. Our findings suggest that addressing these social determinants may be an important component of comprehensive pre-eclampsia prevention strategies, particularly in settings with significant healthcare disparities. High-risk patients identified in the second trimester may benefit from several targeted interventions, including antihypertensive therapy (e.g., labetalol, methyldopa), administration of corticosteroids between 24–34 weeks to promote fetal lung maturity, and magnesium sulfate for seizure prophylaxis in cases of severe hypertension. Enhanced monitoring through more frequent visits and fetal growth surveillance is also critical. These strategies can help mitigate the severity of complications even when early aspirin prophylaxis is no longer applicable.

### 4.4. Implementation Framework for Clinical Practice

Based on our findings, we propose the following implementation framework for integrating the risk score into clinical practice across different healthcare settings:

1. Assessment timing: The risk assessment should be optimally performed during routine second-trimester visits (20–24 weeks), although risk scoring can further be applied to any gestational age on first presentation.
2. Risk-stratified management protocols:
  - Low-risk patients (0–4 points): Standard prenatal care with visits every 4–6 weeks, routine blood pressure monitoring, and standard education about pre-eclampsia warning signs.
  - Moderate-risk patients (5–7 points): Increased surveillance with visits every 2–3 weeks, consideration of low-dose aspirin (although less effective when started after 16 weeks, some benefit may still be observed), and optional biomarker testing where available.
  - High-risk patients (≥8 points): Intensive monitoring with weekly or biweekly visits, regular assessment of maternal organ function (liver enzymes, platelet counts, renal function), enhanced fetal surveillance with serial ultrasounds, and planning for possible early delivery after 37 weeks or sooner if complications develop.
3. Resource allocation strategies:
  - In low-resource settings: Focus advanced monitoring resources on high-risk patients while maintaining standard care for low-risk patients.
  - In medium-resource settings: Implement tiered care model with intensity scaled to risk category.
  - In high-resource settings: Integrate second-trimester risk score with first-trimester biomarker screening when available for comprehensive risk assessment.
4. Healthcare provider education: Implement standardized training for clinicians on risk score calculation, interpretation, and management protocols.
5. Patient education materials: Develop risk-appropriate educational resources, emphasizing different warning signs for each risk category.
6. Follow-up protocol: Create structured follow-up schedules based on risk category, with clear escalation pathways when warning signs appear.

This implementation framework is designed to be adaptable across various healthcare settings while maintaining the core risk-stratification approach that optimizes resource allocation and patient outcomes.

Overall, tailored management based on risk categorization demonstrates potential for personalized care to mitigate adverse outcomes. For low-risk patients, routine monitoring suffices, reducing the clinical burden while maintaining patient safety. For moderate-risk patients, early intervention with low-dose aspirin, initiated by 16 weeks, has been validated as an effective preventive measure, reducing the likelihood of pre-eclampsia onset [24]. High-risk patients benefit from intensive monitoring and fetal surveillance, with frequent check-ups and biomarker assessments to detect early signs of disease progression. This stratified approach aligns with the literature indicating that aspirin and close monitoring are beneficial in preventing escalation in high-risk pregnancies [25].

### 4.5. Recommendations for Clinical Practice and Future Research

While the model offers a practical tool for second-trimester risk assessment, future studies should focus on increasing its sensitivity to capture a higher percentage of true pre-eclampsia cases. Incorporating additional clinical parameters, such as body mass index (BMI) or early pregnancy uric acid levels, could potentially enhance the model’s predictive capability. BMI, for instance, has been independently associated with an elevated risk of pre-eclampsia and may serve as a valuable addition to the risk stratification tool [3].

In managing pre-eclampsia risk, tailored interventions based on risk stratification are essential to optimize outcomes. For low-risk patients (scores 0–4), standard prenatal care generally suffices, with regular visits scheduled every 4–6 weeks, alongside routine blood pressure monitoring. Patient education remains critical, encouraging early reporting of symptoms like sudden swelling, headaches, or visual disturbances, which allows for prompt intervention if pre-eclampsia signs arise [25,26].

For those in the moderate-risk group (scores 5–7), increased vigilance is required. Prenatal visits are recommended every 2–3 weeks to enable early detection of emerging hypertension or proteinuria, and low-dose aspirin (150 mg) should be initiated by 16 weeks of gestation. While low-dose aspirin is known to reduce pre-eclampsia incidence when initiated by 16 weeks, its efficacy diminishes significantly if started later in pregnancy [25]. Studies have demonstrated that aspirin effectively reduces pre-eclampsia onset in moderate-risk cases by improving placental function, particularly when started early [16]. Optional biomarker screening, such as the sFlt-1/PlGF ratio where feasible, provides additional predictive support and may help track risk progression [27].

High-risk patients (scores ≥ 8) require intensive monitoring, given their increased likelihood of developing severe pre-eclampsia or complications like eclampsia and HELLP syndrome. For these patients, weekly or biweekly visits are advised, with regular assessments of liver enzymes, platelet counts, and renal function to track signs of organ involvement [26]. Fetal surveillance, including serial ultrasounds and non-stress tests in the third trimester, is crucial to monitor placental function and fetal well-being. Early delivery planning around 37 weeks or sooner is recommended if signs of fetal distress or maternal complications arise. Antihypertensive therapy, with agents like methyldopa or labetalol, may be necessary to manage severe hypertension in this group, balancing maternal and fetal safety [12].

These risk-specific management protocols highlight the importance of personalized prenatal care in pre-eclampsia prevention.

In managing pre-eclampsia for pregnancies beyond 20 weeks, where preventive measures like early aspirin initiation are no longer effective, the focus shifts to close monitoring and symptomatic treatment to control blood pressure and mitigate complications.

Antihypertensive medications play a central role in preventing severe complications. Methyldopa, known for its safety in pregnancy, is commonly used for chronic hypertension but acts slowly, making it less suitable for acute cases. Labetalol, with combined beta- and alpha-blocking effects, is effective for both chronic and immediate blood pressure control and can be administered orally or intravenously in urgent situations [12]. Nifedipine, a calcium channel blocker, offers rapid blood pressure reduction and is often used for acute management, though it requires monitoring to avoid excessive drops.

For cases of severe pre-eclampsia with a heightened risk of eclampsia, magnesium sulfate is the preferred treatment to prevent seizures. Administered initially as a 4–6 g IV loading dose followed by maintenance infusions, magnesium sulfate significantly reduces the risk of eclamptic events but requires monitoring for potential toxicity [28].

In high-risk pregnancies where early delivery may be necessary, corticosteroids are administered between 24 and 34 weeks to enhance fetal lung maturity, reducing respiratory complications for preterm neonates. Betamethasone or dexamethasone is typically used, supporting neonatal outcomes in cases of preterm delivery [29].

These targeted medical interventions enable clinicians to manage symptoms effectively in later-presenting pregnancies, balancing maternal and fetal safety and preparing for early delivery when indicated. This approach provides an evidence-based framework for optimizing care in high-risk pre-eclampsia cases.

Further validation across diverse populations and healthcare settings will strengthen the model’s applicability and generalizability. Large-scale, multicenter trials incorporating patients from various demographic backgrounds could provide insights into how sociodemographic and environmental factors influence pre-eclampsia risk and model accuracy. Additionally, integrating novel biomarkers with clinical data might improve early prediction without compromising the model’s accessibility for low-resource settings.

In future studies, we plan to apply the pre-eclampsia risk score to categorize patients into low, moderate, and high-risk groups, guiding personalized follow-up care over a 12-month period. This approach will be compared to standard prenatal care in a control group to assess its impact on postpartum maternal and neonatal outcomes. Regular follow-up assessments at 6 weeks, 3 months, 6 months, and 12 months postpartum will help evaluate maternal recovery and neonatal health, aiming to demonstrate the effectiveness of this risk-based strategy.

### 4.6. Limitations

This study’s retrospective nature and limited scope to a single center may constrain the model’s generalizability to broader populations. Future prospective studies could address these limitations by validating the model in a larger, more heterogeneous population. Another limitation lies in the exclusion of genetic and first-trimester biochemical markers, which are recognized for their predictive strength but were beyond the scope of this study. While the model’s simplicity and reliance on clinical factors ensure ease of use in low-resource settings, incorporating advanced markers when available would likely enhance its precision in predicting pre-eclampsia.

Additionally, the matching process for controls focused primarily on age and delivery timing but did not account for all potential confounding variables. This may have introduced some selection bias that could influence the model’s performance in real-world settings.

Additionally, outpatient data on low-dose aspirin usage and smoking history were not consistently available and could not be included in the final model. While gestational age was originally part of the score, it was excluded in the final version due to concerns of circularity. These factors may influence the completeness of the model and should be considered in future prospective studies.

Finally, while our model demonstrated good calibration in our study population, the performance metrics may vary in populations with different baseline pre-eclampsia prevalence rates. This highlights the importance of local validation before widespread implementation.

## 5. Conclusions

In this study, we developed and validated a novel risk score for assessing pre-eclampsia in pregnancies presenting after 20 weeks. This risk score enables stratification of patients into low, moderate, and high-risk categories using straightforward clinical data, addressing a critical need for practical risk assessment tools in diverse healthcare settings. The model demonstrated high specificity (97.8%) and strong predictive capabilities (AUC 0.91), making it a reliable addition to prenatal care, particularly where access to first-trimester biomarker screenings is limited.

Our findings underscore the value of risk-based management in improving maternal and neonatal outcomes through targeted monitoring and timely interventions. Significant differences in outcomes were observed across risk categories, with high-risk patients experiencing substantially higher rates of maternal complications (eclampsia 12.3% vs. 0% in low-risk) and poorer neonatal outcomes (mean birth weight difference of 486 g between high and low-risk groups).

The risk score facilitates personalized care, optimizing resource use and enabling healthcare providers to prioritize patients at the highest risk for adverse outcomes. It is particularly valuable in addressing healthcare disparities, as socioeconomic factors like education level and insurance status were shown to significantly impact pre-eclampsia risk and outcomes.

Future studies should aim to enhance the model’s sensitivity, incorporate additional clinical variables, and validate its utility across varied populations. This will ensure broader applicability and reinforce its role in advancing equitable and effective prenatal care to mitigate pre-eclampsia’s impact on maternal and fetal health. Implementation of this risk-stratified approach has the potential to significantly improve outcomes even in settings where early intervention opportunities have been missed.

## Figures and Tables

**Table 1:** Baseline characteristics of study participants.

	Pre-Eclampsia Group (*n* = 350)	Control Group (*n* = 350)	*p*-Value
**Maternal age (years)**			
<30	92 (26.3%)	169 (48.3%)	0.0003
30–35	163 (46.6%)	146 (41.7%)	0.182
>35	95 (27.1%)	35 (10.0%)	<0.0001
**Gestational age at delivery (weeks)**	36.2 ± 2.8	39.1 ± 1.4	<0.0001
**Parity**			
Nulliparous	198 (56.6%)	142 (40.6%)	0.0002
Multiparous	152 (43.4%)	208 (59.4%)	0.0002
**Pre-existing conditions**			
Chronic hypertension	87 (24.9%)	14 (4.0%)	<0.001
Diabetes mellitus	63 (18.0%)	21 (6.0%)	0.003
**Education level**			
Below high school	126 (36.0%)	63 (18.0%)	0.0007
High school or above	224 (64.0%)	287 (82.0%)	0.0007
**Health insurance**			
Yes	263 (75.1%)	312 (89.1%)	0.0016
No	87 (24.9%)	38 (10.9%)	0.0016
**BMI before pregnancy (kg/m^2^)**	27.3 ± 5.2	24.8 ± 4.1	0.0023

Data are presented as mean ± standard deviation for continuous variables and n (%) for categorical variables. *p*-values were calculated using independent samples *t*-test for normally distributed continuous variables, Mann–Whitney U test for non-normally distributed continuous variables, and Chi-square test or Fisher’s exact test (when expected cell counts < 5) for categorical variables. BMI: Body Mass Index. Chronic hypertension was defined as blood pressure ≥ 140/90 mmHg documented before pregnancy or before 20 weeks of gestation, or ongoing antihypertensive treatment. Diabetes mellitus includes both pre-existing diabetes and gestational diabetes diagnosed before enrollment. Educational level below high school includes primary education and incomplete secondary education. A *p*-value < 0.05 was considered statistically significant.

**Table 2:** Baseline characteristics of study participants.

Outcome	Low Risk (*n* = 382)	Moderate Risk (*n* = 196)	High Risk (*n* = 122)	*p*-Value
**Severe pre-eclampsia**	9 (2.4%)	48 (24.5%)	84 (68.9%)	0.0004
**Eclampsia**	0 (0%)	3 (1.5%)	15 (12.3%)	<0.0001
**HELLP syndrome**	2 (0.5%)	7 (3.6%)	11 (8.7%)	0.0013
**ICU admission**	5 (1.3%)	12 (6.1%)	23 (18.9%)	0.0002
**Emergency cesarean delivery**	76 (19.9%)	72 (36.7%)	87 (71.3%)	<0.0001
**Postpartum hemorrhage**	15 (3.9%)	17 (8.7%)	26 (21.3%)	0.0007
**Antihypertensive therapy required**	14 (3.7%)	63 (32.1%)	103 (84.4%)	<0.0001
**Mean systolic BP (mmHg)**	123.4 ± 10.2	142.6 ± 15.8	157.8 ± 18.3	0.0003
**Mean diastolic BP (mmHg)**	78.2 ± 7.4	88.9 ± 10.2	98.5 ± 11.7	0.0008

Data are presented as mean ± standard deviation for continuous variables and n (%) for categorical variables. *p*-values represent the statistical significance of differences across all three risk groups using one-way ANOVA with post hoc Bonferroni correction for normally distributed continuous variables, Kruskal–Wallis test for non-normally distributed continuous variables, and Chi-square test or Fisher’s exact test for categorical variables. BP: Blood pressure; ICU: Intensive Care Unit; HELLP: Hemolysis, Elevated Liver enzymes, Low Platelets syndrome. Severe pre-eclampsia was defined according to ACOG criteria as systolic BP ≥ 160 mmHg or diastolic BP ≥ 110 mmHg, or evidence of end-organ damage. Emergency cesarean delivery refers to cesarean sections performed due to maternal or fetal indications resulting from pre-eclampsia or related complications. Postpartum hemorrhage was defined as blood loss ≥ 1000 mL within 24 h of delivery. A *p*-value < 0.05 was considered statistically significant. Blood pressure values reflect the highest documented readings at first presentation, when risk was assessed or before antihypertensive therapy was initiated.

**Table 3:** Neonatal outcomes by risk category.

Outcome	Low Risk (*n* = 382)	Moderate Risk (*n* = 196)	High Risk (*n* = 122)	*p*-Value
**Birth weight (g)**	3284 ± 432	3076 ± 526	2798 ± 612	0.0003
**Low birth weight (<2500 g)**	21 (5.5%)	29 (14.8%)	42 (34.4%)	<0.0001
**Head circumference (cm)**	34.2 ± 1.8	33.4 ± 2.1	31.9 ± 2.4	0.0017
**Thorax circumference (cm)**	33.1 ± 1.6	32.3 ± 1.9	30.4 ± 2.2	0.0009
**Fetal length (cm)**	50.2 ± 2.3	48.9 ± 2.7	46.3 ± 3.2	0.0002
**APGAR score (1 min)**	8.6 ± 0.7	8.2 ± 0.9	7.1 ± 1.4	0.0015
**APGAR score (5 min)**	9.3 ± 0.5	8.9 ± 0.8	7.8 ± 1.2	0.0023
**NICU admission**	18 (4.7%)	26 (13.3%)	37 (30.3%)	0.0006
**Preterm birth (<37 weeks)**	28 (7.3%)	42 (21.4%)	61 (50.0%)	<0.0001
**Respiratory distress syndrome**	12 (3.1%)	19 (9.7%)	28 (23.0%)	0.0018

Data are presented as mean ± standard deviation for continuous variables and n (%) for categorical variables. *p*-values represent the statistical significance of differences across all three risk groups using one-way ANOVA with post hoc Bonferroni correction for normally distributed continuous variables, Kruskal–Wallis test for non-normally distributed continuous variables, and Chi-square test or Fisher’s exact test for categorical variables. NICU: Neonatal Intensive Care Unit. Low birth weight was defined as birth weight < 2500 g. APGAR scores were assessed at 1 and 5 min after birth according to standard clinical practice. Preterm birth was defined as delivery before 37 completed weeks of gestation. Respiratory distress syndrome was diagnosed based on clinical and radiological criteria. A *p*-value < 0.05 was considered statistically significant.

**Table 4:** Neonatal outcomes by risk category.

Subgroup	Sensitivity	Specificity	AUC (95% CI)	*p*-Value
**Overall**	74.4%	97.8%	0.91 (0.88–0.94)	<0.0001
**By parity**				
Nulliparous	79.2%	96.5%	0.93 (0.90–0.96)	<0.0001
Multiparous	68.7%	94.2%	0.88 (0.84–0.92)	<0.0001
**By age**				
<35 years	76.3%	95.8%	0.92 (0.89–0.95)	<0.0001
≥35 years	71.5%	93.6%	0.89 (0.85–0.93)	0.0002
**By education**				
Below high school	80.1%	94.5%	0.90 (0.86–0.94)	0.0003
High school or above	72.2%	96.1%	0.91 (0.88–0.94)	0.0005
**By insurance status**				
Insured	73.8%	96.8%	0.91 (0.88–0.94)	0.0002
Uninsured	81.2%	92.3%	0.90 (0.85–0.95)	0.0015

AUC: Area Under the Curve; CI: Confidence Interval. Sensitivity and specificity values were calculated at the optimal cut-off point determined by the Youden index (maximum value of sensitivity + specificity − 1). Model performance was assessed within each demographic subgroup independently. *p*-values represent the statistical significance of the AUC compared to the null hypothesis (AUC = 0.5, no discrimination). The difference in model performance between subgroups was tested using the DeLong method for comparing AUCs from dependent ROC curves. Nulliparous was defined as no previous birth beyond 20 weeks of gestation. Education level “Below high school” includes primary education and incomplete secondary education. All analyses were performed with a significance threshold of *p* < 0.05.

**Table 5:** Comparison with existing pre-eclampsia prediction models.

Model	Timing (Trimester)	Key Components	AUC	Sensitivity	Specificity	Key Advantage	Key Limitation
Current study	2nd	Clinical factors	0.91	74.4%	97.8%	No specialized tests needed	Moderate sensitivity
NICE Guidelines [15]	1st	Maternal history	0.77	89%	61%	Simple, no tests	Low specificity
FMF Bayes [16]	1st	Maternal history, MAP, UTPI, PAPP-A, PlGF	0.95	89%	90%	Highest accuracy	Requires specialized tests
ASPRE Trial [17]	1st	Maternal history, MAP, UTPI, PAPP-A, PlGF	0.92	76%	91%	Validated in large trial	Requires specialized tests
sFlt-1/PlGF [18]	Any	Blood biomarkers	0.89	82%	95%	Good for rule-out	Expensive biomarker test
Espinoza et al. [19]	2nd	Doppler + maternal history	0.85	72%	83%	Good mid-pregnancy tool	Requires Doppler ultrasound

AUC: Area Under the Curve; NICE: National Institute for Health and Care Excellence; FMF: Fetal Medicine Foundation; ASPRE: Aspirin for Evidence-Based Preeclampsia Prevention; MAP: Mean Arterial Pressure; UTPI: Uterine Artery Pulsatility Index; PAPP-A: Pregnancy-Associated Plasma Protein-A; PlGF: Placental Growth Factor; sFlt-1: soluble fms-like tyrosine kinase-1. Model performance metrics were extracted from the cited publications. The “Key Advantage” and “Key Limitation” columns represent the most notable strength and weakness of each model based on literature review and clinical applicability. Early onset pre-eclampsia is defined as pre-eclampsia requiring delivery before 34 weeks of gestation. Sensitivity and specificity values are reported for the prediction of pre-eclampsia at any gestational age unless otherwise specified in the original publications.

## Data Availability

The raw data supporting the conclusions and results of this article will be made available upon request.
